# Associations between childhood autistic traits and adolescent eating disorder behaviours are partially mediated by fussy eating

**DOI:** 10.1002/erv.2902

**Published:** 2022-04-06

**Authors:** Virginia Carter Leno, Nadia Micali, Rachel Bryant‐Waugh, Moritz Herle

**Affiliations:** ^1^ Department of Biostatistics Institute of Psychiatry, Psychology & Neuroscience King's College London London UK; ^2^ Department of Pediatrics, Gynaecology and Obstetrics Faculty of Medicine University of Geneva Geneva Switzerland; ^3^ Department of Psychiatry Faculty of Medicine University of Geneva Geneva Switzerland; ^4^ Great Ormond Street Institute of Child Health University College London London UK; ^5^ Maudsley Centre for Child and Adolescent Eating Disorders South London and Maudsley NHS Foundation Trust London UK; ^6^ Department of Child and Adolescent Psychiatry Institute of Psychiatry, Psychology & Neuroscience King's College London London UK

**Keywords:** ALSPAC, autism, eating disorders, fussy eating, longitudinal analyses

## Abstract

**Objective:**

Previous literature shows an increased risk for eating disorders in autistic individuals. This study tested whether fussy eating contributes to the association between childhood autistic traits and adolescent eating disorder behaviours.

**Method:**

Using data from the Avon Longitudinal Study of Parents and Children, we estimated the intercept and slope of parent‐rated autistic traits and fussy eating between 7 and 14 years (*N* = 8982) and their association with self‐reported eating disorder behaviours at age 14 years, including the indirect path from autistic traits to eating disorder behaviours via fussy eating. Analyses were adjusted for child sex, maternal age at delivery, maternal body mass index and maternal education.

**Results:**

Analyses found a small indirect pathway from autistic traits intercept to eating disorder behaviours via fussy eating slope (*b* = 0.017, 95% CI = 0.002–0.032, *p* = 0.026), with higher levels of autistic traits at age 7 years being associated with a shallower decline in fussy eating, which in turn was associated with greater eating disorder behaviours.

**Conclusion:**

Findings point towards fussy eating as a potential link between childhood autistic traits and later disordered eating. Addressing fussy eating patterns before they become entrenched may decrease risk for eating disorders later in development.

## Highlights

- There is evidence that high levels of autistic traits are associated with an increased likelihood of experiencing an eating disorder.
- The current study sought to test whether the link between autism and eating disorders could in part be explained by variation in fussy eating.
- In a large prospective cohort study, we found a significant indirect effect of fussy eating, such that higher childhood autistic traits were associated with a shallower decline in fussy eating between childhood and adolescence, which in turn was associated with higher levels of eating disorder behaviours in adolescence.

**Abbreviations**
ADHD: Attention‐deficit hyperactivity disorder
ALSPAC: Avon longitudinal study of parents and children
ARFID: Avoidant restrictive food intake disorder
BMI: Body mass index
CFI: Comparative fit index
CIs: Confidence intervals
RMSEA: Root mean square error of approximation
SCDC: Social and communication disorder checklist
SEM: Structural equation model
TLI: Tucker‐Lewis index

## 1 INTRODUCTION

Autism spectrum disorder (henceforth referred to as autism) is a neurodevelopmental condition characterised by difficulties in social functioning, communication, the presence of restricted and repetitive behaviours and sensory anomalies (American Psychiatric Association, 2013). Autism is associated with increased risk for psychopathology as a whole, including eating disorders (Lai et al., 2019). The category of eating disorders includes anorexia nervosa, bulimia nervosa and binge eating disorder, but more recently avoidant restrictive food intake disorder (ARFID) has also been incorporated into this diagnostic category. In general population samples, the prevalence of lifetime diagnosis of any eating disorder is around <10% (but rates are increasing; Galmiche et al., 2019; Lindvall Dahlgren et al., 2017). Although there is limited research specifically examining at the prevalence of eating disorders in autistic people, online self‐report studies find autistic individuals are twice as likely to experience eating disorders compared to their typically developing counterparts (Sedgewick et al., 2021), and heterogeneous outpatient samples of adults with neurodevelopmental diagnoses (primarily autism and attention deficit hyperactivity disorder; ADHD) are characterised by a heightened prevalence of eating disorders (Karjalainen et al., 2016). Furthermore, studies of individuals with eating disorders have suggested that children with early onset eating disorders exhibit increased autism traits (Pooni et al., 2012), and a high prevalence of women with eating disorders may have unrecognised autism (Mandy & Tchanturia, 2015). Similarly, systematic reviews report increased prevalence of autism in eating disorder populations (Westwood & Tchanturia, 2017). However, questions have been raised as to whether being autistic or having high levels of autistic traits is truly a risk factor for the development of eating disorders, as many of the behaviours assumed to be indicative of autism (e.g., behavioural rigidity, inflexibility, social difficulties) could also be a consequence of having an eating disorder (e.g., the impact of starvation on cognition; Katzman et al., 2001) and/or associated other confounders (e.g., obsessive compulsive disorder that often co‐occurs with eating disorders could explain the observed inflexibility; Kaye et al., 2004). To address this question of directionality, two studies have used data from a longitudinal population cohort to examine whether increased autistic traits precede the emergence of eating disorder symptoms (Dinkler et al., 2021; Solmi et al., 2021). These studies suggest that autistic traits (Solmi et al., 2021) in childhood, or associated cognitive difficulties (e.g., difficulties with emotion recognition; Schaumberg et al., 2020), are associated with an increased likelihood of eating disorder behaviours in adolescence. However, others did not find differences in autistic traits at age 9 between individuals who later met diagnostic criteria for anorexia nervosa in adolescence (Dinkler et al., 2021).

Therefore, although there is evidence to suggest that autism may be a risk factor for the development of eating disorders, the mechanisms of effect are unclear. Thematic analysis of interviews with autistic adults with eating disorders have revealed a number of possible mechanisms that could underpin the association between autism and eating disorders, including food‐specific sensory sensitivities (Brede et al., 2020; see also Kinnaird et al., 2019). This hypothesis is supported by meta‐analyses which report that autistic children are five times more likely to have feeding problems (such as chronic food selectivity and food refusal; Sharp et al., 2013), systematic reviews which suggest food selectively is more prevalent in autistic populations (Mari‐Bauset et al., 2014), and studies that indicate autistic adults report higher rates of selective eating compared to typically developing individuals (Kuschner et al., 2015). In particular, autism is thought to often co‐occur with ARFID (Farag et al., 2021), which is characterised by fussy eating, such as persistent patterns of food avoidance or restriction (i.e., limited range of accepted foods, limited overall amount eaten, or both) which negatively affect the nutritional status or physical health of the individual, and/or result in significant psychosocial impairment (American Psychiatric Association, 2013; World Health Organisation, 2019). Although not taken from representative samples, recent studies find around 20% of autistic children are at high risk of ARFID based on their eating behaviours (Koomar et al., 2021). There is some evidence that elevated and persistent fussy eating in childhood is associated with slightly increased risk for anorexia nervosa in adolescence (Herle, Stavola, Hubel, Abdulkadir, et al., 2020; Nicholls & Viner, 2009). Hence, fussy eating might overall be one of the mechanisms between autistic traits in childhood and a risk factor for the development of eating disorders in adolescence. This mechanism is of particular interest, as there is evidence to suggest that fussy eating is amenable to parental intervention in typically developing children (Fildes et al., 2014). Thus, fussy eating may represent a treatable target to promote positive outcomes for autistic youth, including decreasing the likelihood of a child with selective eating developing a clinically significant eating disorder.

In the current paper, we aim to extend previous work on the link between autism and eating disorders to test whether this association is in part driven by fussy eating. We examine the associations between autistic traits, fussy eating, and symptoms of eating disorders between 7 and 14 years in the Avon Longitudinal Study of Parents and Children (ALSPAC) cohort. Fussy eating, here, consists of parental measures of child being very choosy around food, refusing to eat and overall feeding difficulties. These measures have been used to study fussy eating and its association with later BMI and eating disorder outcomes (Herle, Stavola, Hubel, Abdulkadir, et al., 2020; Herle, Stavola, Hubel, Ferreira, et al., 2020). We present results from a pre‐registered analysis (DOI: 10.17605/OSF.IO/3YFRP), where we hypothesised that higher levels of autistic traits at 7 years would be associated with higher levels of fussy eating at 7 years, and greater increase in fussy eating behaviours between 7 and 13 years. We also hypothesised higher levels of autistic traits at age 7 would be associated with increased levels of eating disorder behaviours at 14 years (as has been reported by others in the same cohort; Solmi et al., 2021). We extend previous work by testing the hypothesis that longitudinal trajectories of fussy eating between 7 and 13 years would mediate the observed association between autistic traits at 7 years and eating disorder behaviours at 14 years.

## 2 METHODS

### 2.1 Sample

Participants included in this study are a subsample of adolescents of the population based ALSPAC cohort (Boyd et al., 2013; Fraser et al., 2013). All pregnant women in a defined geographical area in the Southwest of England that were expected to have a child in the period of 1 April 1991 until 31 December 1992 were contacted to participate in the original cohort. At the beginning, 14,451 pregnant women took part, and 13,988 children were alive at the end of year one. To guarantee independence of individuals, one sibling per set of multiple births (*n* = 203 sets) was randomly included in our sample. Please note that the study website contains details of all the data that are available through a fully searchable data dictionary and variable search tool and reference the following webpage: http://www.bristol.ac.uk/alspac/researchers/our‐data/.

Ethical approval for the ALSPAC participants was obtained from the ALSPAC Ethics and Law Committee and the Local Research Ethics Committees: www.bristol.ac.uk/alspac/researchers/research‐ethics/. Consent for biological samples was collected in accordance with the Human Tissue Act (2004).

The subsample for these analyses included participants that had at least one of the measures of autistic traits or fussy eating (*N* = 8982). A breakdown of number of observations available for each variable is presented in Table 1.

**TABLE 1:** Descriptive statistics of the analysis sample taken from the Avon Longitudinal Study of Parents and Children

	Age	N	Median/Mean	IQR/SD
T1 SCDC	7.6 years	7813	2	0‐4
				Range: 0‐24
T2 SCDC	10.7 years	7466	1	0‐3
				Range: 0‐24
T3 SCDC	13.8 years	6843	1	0‐4
				Range: 0‐24
T1 fussy eating	6.8 years	8174	1.58	0.26
T2 fussy eating	8.7 years	7967	1.71	0.43
T3 fussy eating	9.6 years	7868	1.69	0.35
T4 fussy eating	13.1 years	6903	1.47	0.22
Binge eating, *n* scored as present (%)	14.1 years	5876	135 (2.3%)	‐
Fasting, *n* scored as present (%)	14.1 years	6007	176 (2.9%)	‐
Purging, *n* scored as present (%)	14.1 years	6006	42 (0.7%)	‐
Baseline covariates				
Child sex, *n* male (%)	At birth	14,682	7488 (51%)	
Maternal age at birth	At birth	13,778	27.9 years	4.9
				Range: 15‐44
Pre‐pregnancy BMI	12 weeks gestation	11,387	22.9	3.9
				Range: 12.5–54.7
Maternal education, *n* with A‐Levels or above (%)		14,703	6788 (46.2)	‐
Ethnicity	At birth		11,523 White	‐
			613 non‐White	

### 2.2 Measures

#### 2.2.1 Autistic traits

The Social and Communication Disorder Checklist (SCDC) was used by parents to indicate the social communication difficulties of their children when they were 7, 11 and 14 years old (Skuse et al., 2005). The checklist consists of 12 items and asks parents to rate the extent to which these communication and behavioural difficulties apply to their children using a three‐point scale (“not true”, “quite or sometimes true”, “very often true”). The SCDC has been widely used in the literature and validated against other clinical measures for identifying autism diagnostic status and autistic symptoms (Bolte et al., 2011; Pickard et al., 2017). Answers on all 12 items were added into a sum score at each wave using complete cases only.

#### 2.2.2 Fussy eating

Fussy eating was measured by three parent reported questions when the participants were 7, 8, 9 and 13 years old. Questions were: “How worried are you because your child is choosy?”, “How worried are you because your child has feeding difficulties?” and “How worried are you because your child is refusing food?”. Parents used a four‐point Likert scale: “No did not happen”, “Yes this did happen, but I was not worried”, “Yes, I was worried about it a bit” and “Yes, I worried greatly”. The top two categories were collapsed and an average score for fussy eating was calculated for each wave. This measure of fussy eating has previously developed in this sample and used in multiple previous studies (Herle, Stavola, Hubel, Abdulkadir, et al., 2020; Herle, Stavola, Hubel, Ferreira, et al., 2020).

#### 2.2.3 Eating disorder behaviours

Participants answered questions probing the frequency of eating disorder behaviours over the past year when they were 14 years old. The three behaviours in question were: Fasting for weight loss (“During the past year, how often did you fast (not eat for at least a day) to lose weight or avoid gaining weight?”), binge eating (“Sometimes people will go on an “eating binge”, where they eat an amount of food that most people would consider to be very large, in a short period of time. During the past year, how often did you go on an eating binge?”, together with a question asking if participants felt like they lost control during the eating binge)) and purging (“During the past year, how often did you make yourself throw up (vomit) to lose weight or avoid gaining weight?” and “During the past year, how often did you take laxatives to lose weight or avoid gaining weight?”). Response options were: “Never”, “Less than once a month”, “1–3 times a month”, “Once a week”, “2–6 times a week” and “Everyday”. Participants were considered to engage in eating disorder behaviours if they answered “at least 1–3 times a month”. These questions have been extensively used in eating disorder research in this sample, as they cover key behaviours of clinical diagnostic criteria of eating disorders (Micali et al., 2017). These binary indicators were used to construct a continuous latent factor score. All items loaded significantly on this factor (fasting loading = 0.90, binge eating loading = 0.46, purging loading = 0.73) and the model fit was good (χ^2^(1) = 0.39, *p* = 0.53, RMSEA = 0.00 (90% CIs = 0.00–0.03), CFI/TLI = 1.00).

#### 2.2.4 Covariates

The covariates included were child sex, maternal age at delivery, maternal body mass index prior to pregnancy, and maternal education at birth of the child (dichotomised as having completed education up to A‐Levels, the usual requirement for applying to university in the UK (yes/no)).

## 3 ANALYSES

**FIGURE 1:**
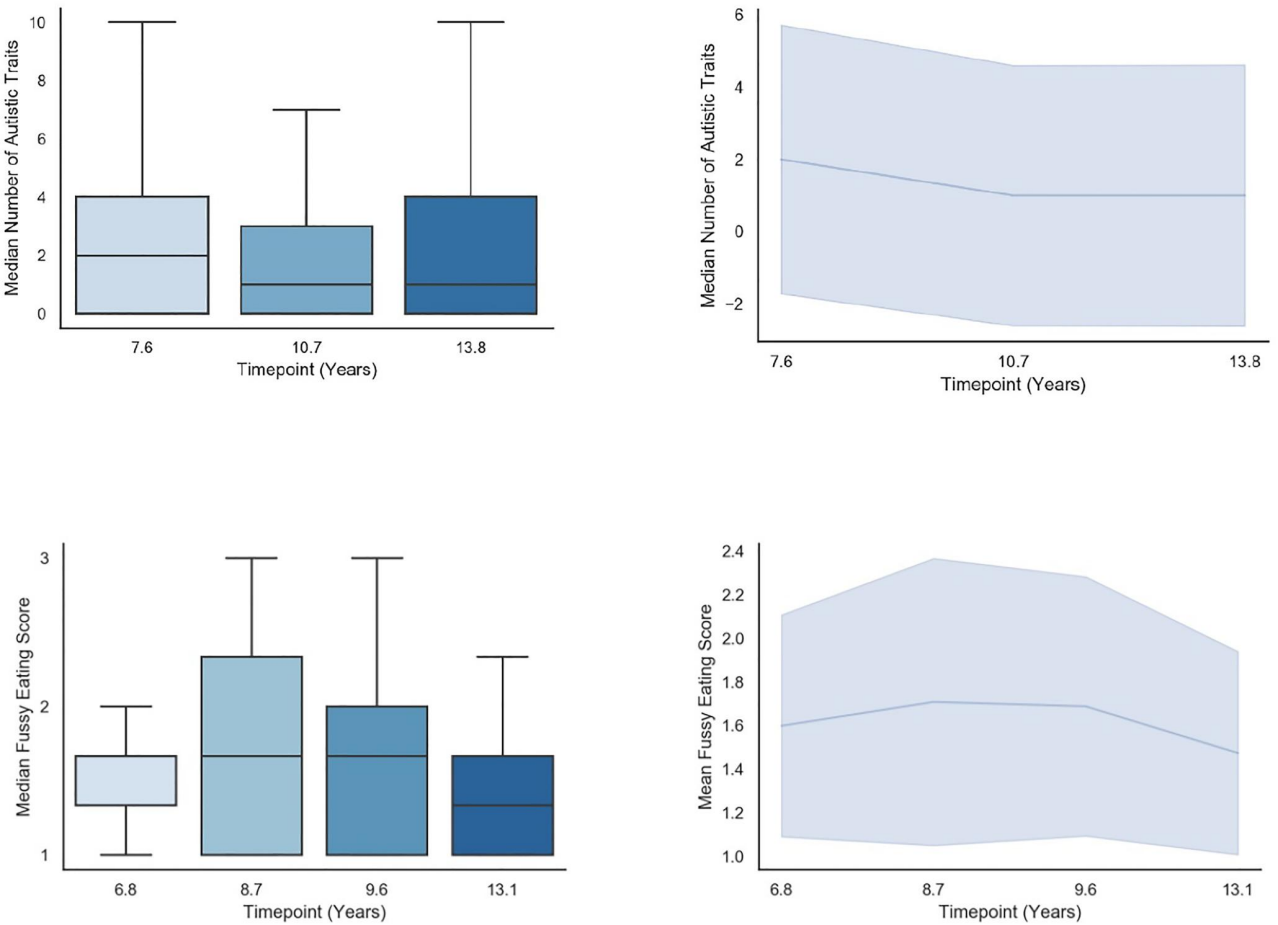
Descriptive plots of autistic traits and fussy eating at each wave and across time

Analyses were conducted in two steps. First, latent factors of intercept and slope were estimated for repeated measures of autistic traits and fussy eating separately in Mplus. Higher order polynomial terms, such as quadratic and cubic slopes were not considered for the autistic trait measures as these require more timepoints to be adequately fitted to the data. For fussy eating we fitted quadratic term, however this specification resulted in a non‐positive definite covariance matrix, driven by a correlation greater or equal to one between the linear and quadratic term. Hence, we decided to not to include a quadratic term as the information contained within in was not adding any extra information over the linear term. Due to the relative rarity of individuals with high levels of autistic traits (see Figure 1), scores were treated as count data and a negative binomial estimator was used. For the fussy eating data, the maximum likelihood robust estimator was used to correct for non‐normal distributions. Individual factor scores for intercept and slope were extracted. Next, these variables were entered into a structural equation model (SEM), with paths from autistic traits/fussy eating to autistic traits/fussy eating slope (i.e., testing whether differences in these variables at age 7 predicted the slope of change between 7 and 13 years, both within and between constructs) and paths from all latent variables to the latent eating disorder behaviours factor at 14. To adjust for relevant confounders, we specified paths from covariates to all other variables in the model. From these SEM models we used the nlcom command to estimate the indirect path from autistic traits intercept to eating disorder behaviours at 14 via the slope of fussy eating (i.e., the a × b path). If main effects were found, three subsequent follow‐up models were run, where each eating disorder behaviour (dieting/fasting, binging, and purging) was treated as a separate outcome (results displayed in Figure S1). As participants dropped out of the study with time, and hence less data is available on the outcome measure, models were estimated using maximum likelihood to account for missing data missing at random. We conducted sensitivity analyses only including participants who had complete information available on all variables in the model to check results were not driven by attrition (*N* = 3026, see Table S1). We diverged from our pre‐registered protocol (DOI: 10.17605/OSF.IO/3YFRP) in the following ways. First, we had previously stated we would include measurement of autistic traits at age 16 in our estimation of the slope of autistic traits, however we only used measurements at age 7, 11 and 14 to avoid measurement beyond our outcome of eating disorder behaviours. Second, we had specified we would run multi‐group models to examine sex‐specific patterns of association, however, given the low numbers of males with eating disorder behaviours, sex‐specific models failed to converge. Analysis scripts can also be found with our pre‐registered protocol.

## 4 RESULTS

Descriptive statistics of the sample can be found in Table 1. Distribution of autistic trait and fussy eating variables at each time point, and the overall mean change over time is shown in Figure 1.

**FIGURE 2:**
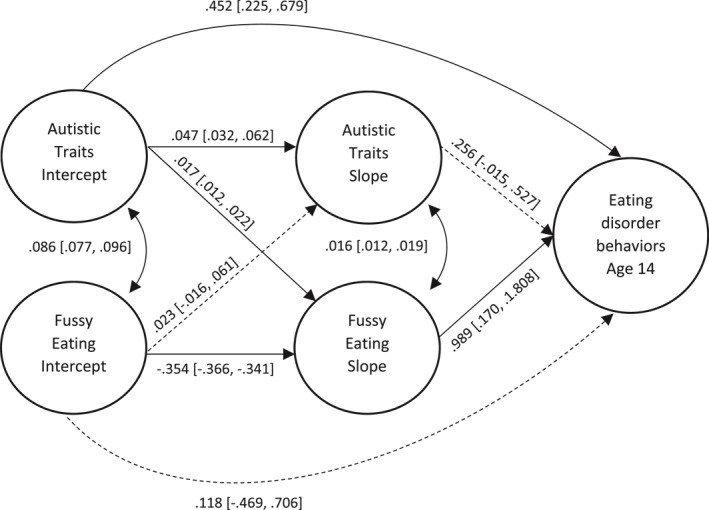
Path estimates [95% confidence intervals] for the full model

Fussy eating declined throughout development (indicated by a negative slope), whereas autistic traits remained relatively stable throughout. Path estimates for the SEM models are listed in Table 2, and results are illustrated in Figure 2. Autistic traits at age 7 were associated with the slope of fussy eating between 7 and 13 years (*b* = 0.017, 95% CI = 0.012–0.022, *p* < 0.001), such that higher autistic traits at 7 were associated with less reduction in fussy eating over time. Autistic traits at age 7 were also associated with eating disorder behaviours at 14 (the direct effect; *b* = 0.452, 95% CI = 0.225–0.679, *p* < 0.001), with higher traits associated with higher latent scores of eating disorder behaviours. The slope of fussy eating between 7 and 13 years was associated with eating disorder behaviours (*b* = 0.989, 95% CI = 0.170–1.808, *p* = 0.018), such that less reduction in fussy eating was associated with higher latent scores of eating disorder behaviours. The indirect pathway from autistic traits intercept to eating disorder behaviours via fussy eating was small but statistically significant (*b* = 0.017, 95% CI = 0.002–0.032, *p* = 0.026). We calculated the proportion mediated by the indirect effect: (indirect effect/(indirect effect + direct effect) * 100). Results suggest that around 4% (95% CIs = 1%–7%) of the association between autistic traits at age 7 and eating disorder behaviours at age 14 could be accounted for by the mediating effect of fussy eating. Results from follow‐up models, examining effects for binge eating, fasting and purging independently, are also shown in Figure S1. Tests of mediation effects found the indirect pathway from autistic traits at age 7, via the slope of fussy eating, was significant for binge eating (*b* = 0.020, 95% CI = 0.006–0.035, *p* < 0.001), but not for fasting or purging (*p* = 0.179, *p* = 0.166 respectively). Results from sensitivity analyses including complete‐case records were comparable to the main analyses (see Table S1).

**TABLE 2:** Path estimates for full mediation structural equation model, *N* = 8982

	Coefficient	95% confidence Intervals		*p* value
Autistic traits intercept				
Sex	−0.203	−0.247	−0.158	<0.001
Maternal BMI	0.000	−0.006	0.006	0.969
Maternal age at delivery	−0.008	−0.013	−0.003	0.003
Maternal education	−0.050	−0.097	−0.003	0.037
Fussy eating intercept				
Sex	−0.029	−0.046	−0.012	0.001
Maternal BMI	−0.002	−0.004	0.001	0.132
Maternal age at delivery	0.007	0.005	0.009	<0.001
Maternal education	0.021	0.002	0.039	0.027
Autistic traits slope				
Fussy eating intercept	0.023	−0.016	0.061	0.247
Autistic traits intercept	0.047	0.032	0.062	<0.001
Sex	0.056	0.025	0.088	<0.001
Maternal BMI	0.005	0.000	0.009	0.030
Maternal age at delivery	−0.002	−0.005	0.002	0.274
Maternal education	0.014	−0.019	0.046	0.410
Fussy eating slope				
Fussy eating intercept	−0.354	−0.366	−0.341	<0.001
Autistic traits intercept	0.017	0.012	0.022	<0.001
Sex	0.001	−0.009	0.011	0.855
Maternal BMI	0.000	−0.001	0.002	0.590
Maternal age at delivery	0.000	−0.001	0.001	0.735
Maternal education	−0.009	−0.019	0.002	0.118
Eating disorder behaviours at 14				
Fussy eating intercept	0.118	−0.469	0.706	0.693
Autistic traits slope	0.256	−0.015	0.527	0.064
Autistic traits intercept	0.452	0.225	0.679	<0.001
Fussy eating slope	0.989	0.170	1.808	0.018
Sex	2.213	1.369	3.057	<0.001
Maternal BMI	0.034	−0.021	0.089	0.225
Maternal age at delivery	−0.026	−0.072	0.021	0.283
Maternal education	−0.343	−0.787	0.101	0.130

## 5 DISCUSSION

The current paper sought to test a putative mechanism, the course of fussy eating over late childhood and early adolescence, that could explain the association between autistic traits and disordered eating. Results supported our pre‐registered hypotheses, indicating that higher autistic traits at age 7 were associated with less reduction in fussy eating between age 7–13 years, and a lower reduction in fussy eating was associated with higher levels of disordered eating at age 14. Mediation analyses found a small but significant indirect pathway from autistic traits to disordered eating via the slope of fussy eating. Follow‐up analyses suggested this indirect pathway to disordered eating was largely driven by binge eating.

The current paper extends previous reports of an association between autistic traits and disordered eating in the same cohort (Solmi et al., 2021), by testing factors that could explain this observed association. In the whole sample, fussy eating declined between ages 7–13 (as is reported elsewhere; Cardona Cano et al., 2015), however, this decline was shallower (i.e., a lower reduction) in children who had higher levels of autistic traits at age 7. This is in line with research reporting co‐occurrence between fussy eating, ARFID, and autistic traits in clinical and population samples (Kambanis et al., 2020; Sharp et al., 2013; Wallace et al., 2018) and the stability of selective eating behaviours between childhood and adolescence in autistic youth (Bandini et al., 2017). Current results suggested that children with high levels of autistic traits are more likely to continue to have feeding difficulties into early adolescence, and in turn these feeding difficulties increase the likelihood of disordered eating in adolescence. Therefore, results suggest that leaving concerning eating behaviours such as fussy eating unaddressed could lead to greater risk of disordered eating in adolescence (as is found in typically developing youth; Herle, Stavola, Hubel, Abdulkadir, et al., 2020), in addition to other negative consequences of a severely restricted diet, for example, nutritional deficiencies such as iron and zinc (Taylor & Emmett, 2019). Findings emphasise the importance of early intervention to prevent maladaptive feeding patterns becoming entrenched and to potentially decrease the risk for eating disorders later in development. Whilst interventions for fussy eating have been developed in typically developing populations, whether they have similar efficacy in autistic children is not well known (Fildes et al., 2014). One evidence‐based parent‐led intervention focuses on repeated exposure to increase the intake of one specific target vegetable (e.g., broccoli). Results from randomised control trials suggested that after 14 days of exposure to the target vegetable, and using stickers as rewards, the intake increased significantly in comparison to the control group (Fildes et al., 2014). Since then, two interventions have been developed to specifically target eating problems in autistic children combining repeated exposure, parental nutrition education and meal plans (Kuschner et al., 2017; Sharp et al., 2019), and one has been evaluated, where it was deemed feasible with promising preliminary results (Sharp et al., 2019). However, it should be acknowledged that despite statistical significance, the coefficient for the indirect effect from autistic traits to eating disorder behaviours via fussy eating was small (even at the higher bound of the confidence interval), which should be held in mind when evaluating the impact of higher levels of autistic traits on eating disorder outcomes.

Better targeted intervention requires a more fine‐grained theoretical model of why autistic children are more likely to have fussy eating or eating problems. Recent work has implicated restrictive and repetitive behaviours and sensory processing difficulties as potentially being important mechanisms in the aetiology of eating difficulties in autistic children. Analyses of a population‐based cohort reported higher levels of cognitive rigidity at age 4 was associated with a lower BMI in girls, and more restrictive eating in both boys and girls at age 9 (Steegers et al., 2021). In young autistic children (aged 3–10 years), higher levels of sensory sensitivity across all modalities (e.g., tactile, visual, auditory, olfactory, gustatory) are associated with greater eating problems (Nadon et al., 2011). These studies suggest that interventions for eating difficulties in autistic children should also consider the role of core autism characteristics in contributing to and maintaining eating difficulties, as these may need to be factored into support offered, in addition to food exposure.

Contrary to our pre‐registered hypotheses, we did not find strong effects for fasting, but instead post‐hoc models indicated that effects on the disordered eating variable were likely driven by binge eating behaviour. As this hypothesis was not predicted a priori, and it is known that there is strong co‐occurrence between different types of disordered eating (Eddy et al., 2008), we suggest further research is needed to investigate specificity of effects. However, one potential hypothesis is that children with higher autistic traits and fussy eating habits might have a diet rich in fats and carbohydrates (similar to as in found in typical developing children with restrictive or fussy eating behaviours). When paired with sensory/sensation seeking behaviours (known to be more prevalent in autistic youth; Ben‐Sasson et al., 2009), this could lead to overconsumption of certain “safe foods”. Overconsumption of specific foods may also function as a coping strategy for autistic youth when faced with stressful or arousing situations (e.g., those experienced during adolescence when social demands and responsibilities increase).

Strengths of the current work include use of a population cohort with repeated assessments between 7 and 14 years, a combination of parent and self‐report to avoid shared method variance and pre‐specification of hypotheses and analytic strategy. Although many studies have highlighted the heightened prevalence of eating disorders in autistic individuals, to our knowledge few have tested mechanisms that could explain this increase in risk. This is a crucial step to identifying targets for intervention, which in turn will promote positive outcomes in autistic youth.

In terms of limitations, despite a large sample size, the low prevalence of high scorers in both the predictor (autistic traits) and outcome (disordered eating) may have limited our statistical power. Given this low prevalence, and the substantial sex ratio for each phenotype, we were unable to robustly investigate sex differences. As anorexia nervosa appears to be particularly prevalent in autistic women (Brown & Stokes, 2020), future work should investigate whether the proposed mechanism is comparable between autistic men and women. We also relied solely on parent report for assessment of fussy eating (ARFID was not clinically defined when these data were collected). This may have underestimated the true level of fussy eating at the later timepoints when children were moving into adolescence and becoming more independent with their food choices. More precise measurement of eating patterns may help better understand the clinically relevant co‐occurrence of autism and eating difficulties – for example, new psychometric tools for measuring ARFID are now available (Bryant‐Waugh et al., 2019) – and could be used in future observational studies. We also highlight that although our measurement of fussy eating preceded our outcome of disordered eating, they were close together in time and so unmeasured bidirectional associations could be present. Future work should incorporate repeated measurements of fussy and disordered eating with robust methods for assessing directionality of associations (e.g., random intercept cross‐lag panel models; Hamaker et al., 2015) to better understand pathways between the two domains. We used a measure of autistic traits rather than diagnostic status, which may in part explain the relatively small association between autistic traits at age 7 and trajectories of fussy eating, as population‐based cohorts such as the one used presently will have limited numbers of participants with high levels of autistic traits (i.e., individuals who would meet threshold for a clinical diagnosis). However, genetic studies suggest that aetiological influences on autism traits at the extremes are shared with aetiological underpinnings of traits in general population (Robinson et al., 2011), supporting the relevance of findings from general population cohorts for individuals with a clinical diagnosis of autism. The sample of this study consisted of predominately White British children, and future research needs to incorporate cultural differences. This is important, as previous research has indicated that parents with different cultural backgrounds rate the fussiness of their children differently and employ different parental feeding strategies (Blissett & Bennett, 2013; Gu et al., 2017). Finally, it should be noted that although autistic traits predicted the change in fussy eating behaviours over time, it could be that overlapping genetic influences explain the association between these two constructs (e.g., pleiotropy). Twin research has been suggestive of genetic correlations between eating problems and autism in childhood (Lundin Remnelius et al., 2021), however, genome‐wide analyses have not found a genetic correlation between autism and anorexia nervosa (Brainstorm et al., 2018). Binge eating has been genetically linked to anxiety and ADHD, however, although the genetic correlation with autism was positive, this did not reach statistical significance (Hubel et al., 2021), leaving scope for future investigation. New research is needed which incorporates genetic liability for autism and fussy eating into longitudinal designs could explore this hypothesis.

## CONFLICTS OF INTEREST

The authors have reported no biomedical financial interests or potential conflicts of interest.

### DATA AVAILABILITY STATEMENT

The data that support the findings of this study are available on request from the corresponding author. The data are not publicly available due to privacy or ethical restrictions.

## Supporting information

Supporting Information S1
