# Supplementary material for: Associations between childhood autistic traits and adolescent eating disorder behaviours are partially mediated by fussy eating
Source: Eur Eat Disord Rev. 2022 Apr 6;30(5):604–15. doi: 10.1002/erv.2902 (PMC9542277; doi:10.1002/erv.2902)
Supplement: Supplementary file 1 — Supporting Information S1 [file ERV-30-604-s001.docx]

**Supplementary Materials**

**Supplementary Table 1**. Path Estimates for Full Mediation Structural Equation Model in Complete Case Analyses (N=3,026)

|  |  | Coefficient | 95% Confidence  Intervals | | p value |
| --- | --- | --- | --- | --- | --- |
| Autistic Traits Intercept | | | | | |
|  | **Sex** | **-0.147** | **-0.222** | **-0.072** | **0.000** |
|  | Maternal BMI | -0.001 | -0.012 | 0.010 | 0.847 |
|  | Maternal Age at Delivery | -0.003 | -0.012 | 0.006 | 0.508 |
|  | Maternal Education | -0.020 | -0.097 | 0.057 | 0.611 |
| Fussy Eating Intercept | |  |  |  |  |
|  | Sex | -0.025 | -0.055 | 0.005 | 0.097 |
|  | Maternal BMI | -0.002 | -0.006 | 0.003 | 0.445 |
|  | **Maternal Age at Delivery** | **0.010** | **0.006** | **0.014** | **0.000** |
|  | Maternal Education | 0.021 | -0.010 | 0.051 | 0.188 |
| Autistic Traits Slope | |  |  |  |  |
|  | **Fussy Eating Intercept** | **0.073** | **0.005** | **0.142** | **0.036** |
|  | **Autistic Traits Intercept** | **0.040** | **0.013** | **0.067** | **0.004** |
|  | **Sex** | **0.098** | **0.043** | **0.154** | **0.001** |
|  | Maternal BMI | 0.001 | -0.007 | 0.008 | 0.879 |
|  | Maternal Age at Delivery | -0.002 | -0.009 | 0.005 | 0.562 |
|  | Maternal Education | 0.056 | -0.001 | 0.114 | 0.053 |
| Fussy Eating Slope | |  |  |  |  |
|  | **Fussy Eating Intercept** | **-0.368** | **-0.388** | **-0.347** | **0.000** |
|  | **Autistic Traits Intercept** | **0.012** | **0.004** | **0.020** | **0.004** |
|  | Sex | 0.012 | -0.005 | 0.028 | 0.168 |
|  | Maternal BMI | 0.001 | -0.001 | 0.003 | 0.459 |
|  | Maternal Age at Delivery | -0.001 | -0.003 | 0.001 | 0.558 |
|  | Maternal Education | -0.009 | -0.026 | 0.008 | 0.300 |
| Eating Disorder Behaviors at 16 | |  |  |  |  |
|  | Fussy Eating Intercept | 0.628 | -0.180 | 1.436 | 0.128 |
|  | Autistic Traits Slope | 0.295 | -0.053 | 0.642 | 0.097 |
|  | **Autistic Traits Intercept** | **0.307** | **0.021** | **0.592** | **0.035** |
|  | **Fussy Eating Slope** | **2.149** | **0.869** | **3.430** | **0.001** |
|  | **Sex** | **2.131** | **1.084** | **3.179** | **0.000** |
|  | Maternal BMI | 0.049 | -0.028 | 0.126 | 0.210 |
|  | Maternal Age at Delivery | 0.026 | -0.041 | 0.093 | 0.448 |
|  | Maternal Education | -0.276 | -0.858 | 0.306 | 0.352 |

**Supplementary Figure 1.** Path estimates (95% confidence intervals) for each domain of eating disorder behaviors

.26 [.09 - .45]

.05 [-.46 - .56]

.02 [.01 - .02]

.02 [-.01-.06]

.05 [.03 - .06]

-.35 [-.37 - -.34]

.09 [.08 - .10]

.02 [.01 - .02]

.49 [-.21 - .1.18]

.12 [-.11 - .34]

Fasting (present/absent)

.26 [.05 - .48]

.41 [-.16 - .98]

.02 [.01 - .02]

.02 [-.01-.06]

.05 [.03 - .06]

-.35 [-.37 - -.34]

.09 [.08 - .10]

.02 [.01 - .02]

1.18 [.40 – 1.95]

.26 [-.00 - .52]

Bingeing (present/absent)

.26 [.05 - .48]

-.13 [-1.15 - .90] .98]

.02 [.01 - .02]

.02 [-.01-.06]

.05 [.03 - .06]

-.35 [-.37 - -.34]

.09 [.08 - .10]

.02 [.01 - .02]

.98 [-.38 – 2.35]

.17 [-.27 - .61]

Purging (present/absent)
